# A Tetrameric Peptide Derived from Bovine Lactoferricin Exhibits Specific Cytotoxic Effects against Oral Squamous-Cell Carcinoma Cell Lines

**DOI:** 10.1155/2015/630179

**Published:** 2015-11-02

**Authors:** Víctor A. Solarte, Jaiver E. Rosas, Zuly J. Rivera, Martha L. Arango-Rodríguez, Javier E. García, Jean-Paul Vernot

**Affiliations:** ^1^Cellular and Molecular Physiology Group, Biomedical Research Institute, Faculty of Medicine, Universidad Nacional de Colombia, Bogotá 111321, Colombia; ^2^Department of Pharmacy, Faculty of Sciences, Universidad Nacional de Colombia, Bogotá 111321, Colombia; ^3^Department of Chemistry, Faculty of Sciences, Universidad Nacional de Colombia, Bogotá 111321, Colombia; ^4^Centro de Medicina Regenerativa, Faculty of Medicine, Clínica Alemana, Universidad del Desarrollo, 7690000 Santiago, Chile

## Abstract

Several short linear peptides derived from cyclic bovine lactoferricin were synthesized and tested for their cytotoxic effect against the oral cavity squamous-cell carcinoma (OSCC) cell lines CAL27 and SCC15. As a control, an immortalized and nontumorigenic cell line, Het-1A, was used. Linear peptides based on the RRWQWR core sequence showed a moderate cytotoxic effect and specificity towards tumorigenic cells. A tetrameric peptide, LfcinB(20–25)_4_, containing the RRWQWR motif, exhibited greater cytotoxic activity (>90%) in both OSCC cell lines compared to the linear lactoferricin peptide or the lactoferrin protein. Additionally, this tetrameric peptide showed the highest specificity towards tumorigenic cells among the tested peptides. Interestingly, this effect was very fast, with cell shrinkage, severe damage to cell membrane permeability, and lysis within one hour of treatment. Our results are consistent with a necrotic effect rather than an apoptotic one and suggest that this tetrameric peptide could be considered as a new candidate for the therapeutic treatment of OSCC.

## 1. Introduction

Oral squamous-cell carcinoma (OSCC) is fatal in approximately 50% of the diagnosed cases [1]. It can be controlled during the early stages of the disease; however, it is considered to be of poor prognosis with low survival rate in advanced stages (12% on average) [2–4]. The conventional therapeutic methods used for oral cancers—surgery, radiotherapy, and chemotherapy—are quite aggressive and ablative for the patient and can induce notable side effects [5–7]. The occurrence of complications and the limited success of these therapies have aroused great interest in understanding OSCC physiopathology, which may lead to the improvement of current treatments and the development of new therapeutic approaches [8–10].

Lactoferrin (Lf) is an 80 kDa member of the transferrin family of iron-binding glycoproteins, produced and released by neutrophils. Lf is found in mammalian exocrine secretions such as breast milk, saliva, tears, nasal and bronchial mucus, cervical mucus, and seminal fluid [11, 12]. Several biological properties have been attributed to Lf, including antimicrobial, antitumoral, antimetastatic, and anti-inflammatory activities [12]. In particular, Lf anticancer effects have been evaluated for different types of cancer using both* in vitro* and* in vivo* models [13–21], whereby it was determined that bovine Lf (LfB) exhibits greater cytotoxic activity than human Lf (LfH) [22]. In breast and in head and neck cancers, it has been reported that LfB inhibits cell proliferation by arresting cancer cells in the G1-G0 phase of the cell cycle and by increasing the expression of proinflammatory and immune cytokines [19, 20]. McKeown et al. reported that LfB induces cell death in carcinoma cell lines, but not in normal cells, thus evidencing its specificity towards tumoral cells [23]. It has been demonstrated that LfB is able to prevent the development of various types of epithelial cancer (esophageal, tongue, lung, liver, and colon) and metastasis [24, 25]. It has also been shown that LfB activates, through different caspases, the intrinsic and extrinsic pathways of apoptosis in colon cancer and leukemia cells [26, 27]. Furthermore, this protein is able to induce FasL expression by activating the extrinsic pathway of apoptosis and also to inhibit angiogenesis, an important step in tumorigenesis [26, 28].

LfB digestion by the gastric pepsin gives rise to bovine lactoferricin (LfcinB), a cyclic peptide fragment of 25 amino acids (FKCRRWQWRMKKLGAPSITCVRRAF (17–41)) located in the amino-terminal portion of the protein and apparently responsible for its antimicrobial and anticancer effects [10, 29–32]. It has been reported that LfcinB exhibits selectivity towards cancer cells, with cytotoxic activity against different types of cancer cells, including leukemia, fibrosarcomas, melanomas, and colon cancer, without affecting normal fibroblasts, lymphocytes [10, 33–35], or nontransformed cells [36]. It is well known that LfcinB administration inhibits lymphoma, melanoma, and colon carcinoma metastasis to the liver or the lung [33, 37, 38]. These results highlight the potential clinical usefulness of LfcinB in cancer therapy.

LfcinB is highly basic (+8), containing five Arg, three Lys, and some aromatic amino acids (two Trp (W) and two Phe (F)), which confer important amphipathic properties [29]. Natural LfcinB has a cyclic structure formed by a disulfide bond established between the two cysteine residues. It has been suggested that the cyclic form of LfcinB is not required for its antimicrobial [29, 39] or cytotoxic effect* in vitro* in some cancer cell lines [40–42]. Nevertheless, the cyclic structure seems to be required for improving its antitumoral activity, since the linear LfcinB, that is, LfcinB25, exhibits a decreased cytotoxic effect in xenograft models [43]. It is believed that the amphipathic nature of LfcinB allows interaction with the abundant negative charges present in cancer cells, thus contributing to target selectivity [34, 44]. It is thought that the main mechanism of action of LfcinB in tumor cells is cell membrane disruption [43] and activation of the oxidants-, endonucleases-, caspases-, cathepsin B-, and ceramides-dependent apoptotic pathways [40, 45, 46].

The total pepsin hydrolysate of LfB showed a greater growth suppressive effect in leukemic cells and contains peptides derived from the N-terminal portion of the protein. Two short peptides (17-FKCRRWQWRMKKLGAPSITCVR-38 and 17-FKCRRWQWRMKKLGA-31) were identified, both of which exhibit high cytotoxic activity [46]. Moreover, it has been shown that this hydrolysate exhibits antitumor and antimetastatic effects in murine models of cancer [15]. Based on these findings, it has been proposed that the amino acids RRWQWR within these sequences could be the key motif that is the agent of their antitumoral activity. However, the peptide RRWQWR, here called LfcinB(20–25), is ineffective in the leukemic CCRF-CEM and Jurkat cell lines and in the breast carcinoma MDA-MB-231 cell line. Nevertheless, it has been proven that liposomes-encapsulated LfcinB(20–25) induces a clear cytotoxic effect on the Jurkat cell line [40]: the internalized peptide interacts and damages mitochondria by triggering apoptosis [46]. In the present study, we designed new LfcinB25-derived peptides to search for molecules with enhanced cytotoxic effect against OSCC cells.

## 2. Materials and Methods

### 2.1. Protein and Peptides

LfB protein was purchased from Sigma (L9507), while the peptides (Table 1) were synthesized using the SPPS-Fmoc/tBu methodology, as previously reported [47]. Purity of peptides was >90%, determined by RP-HPLC analysis. All peptides had the expected molecular weight, as determined using MS MALDI-TOF.

### 2.2. Cell Lines and Culture Conditions

The cell lines CAL27 (ATCC CRL-2095) and SCC15 (ATCC CRL-1623) and the human immortalized nontumorigenic epithelial esophagus cell line Het-1A (ATCC CRL-2692) were purchased from ATCC (Manassas, VA). CAL27 cells were cultured in Dulbecco's Modified Eagle's Medium (DMEM; Gibco) with 10% of fetal bovine serum (FBS; Gibco). SCC15 cells were cultured in a 1 : 1 mixture of DMEM/F12 medium (Gibco) with 400 ng/mL hydrocortisone and 10% FBS. Het-1A cells were cultured in Bronchial Epithelial Cell Growth Medium (BEBM) with the additives obtained from Lonza/Clonetics Corporation as a kit (CC-3170), without antibiotics. All cells were maintained at 37°C in a 5% CO_2_ humidified atmosphere. For Het-1A cells, the culture flasks were precoated with fibronectin (0.01 mg/mL), bovine collagen type I (0.03 mg/mL), and bovine serum albumin (0.01 mg/mL) dissolved in a culture medium and incubated for 24 h before culturing the cells. Cell stocks were prepared and thawed periodically and used in early subcultures (not exceeding 5–10 population doublings). Cell viability was estimated by Trypan blue exclusion staining and was always higher than 97%. Growth curves for each cell line were done using 3-(4,5-dimethylthiazol-2-yl)-2,5-diphenyltetrazolium bromide (MTT). By this means, the number of cells and the time needed to reach 70% confluence were calculated, allowing cytotoxic evaluation during exponential growth. SCC15 cells having a mesenchymal- or epithelia-like phenotype were separated by their differential adherence to plastic dish and characterized for the expression of specific markers (see Section 2.7).

### 2.3. Cytotoxic Assay

Cytotoxic activity was determined using the MTT assay. Briefly, cells were seeded in 96-well plates with a confluence of approximately 70% (8 × 10^3^ CAL27-cells/well, 4 × 10^3^ SCC15-cells/well, and 1.6 × 10^4^ Het-1A-cells/well). After cell adherence, the culture medium was removed and peptides at different concentrations (between 100 and 6.25 *μ*g/mL) and times (between 1 and 24 h or 48, 72, and 96 h) were added to the wells in the absence of FBS. Staurosporine (STA) was used as positive control (CAL27: 0.4 *μ*g/mL, SCC15 0.6 *μ*g/mL, and Het-1A 0.6 *μ*g/mL). Phase-contrast photomicrographs of the treated cells were taken (Nikon, Eclipse TS500). After treatment, the medium was replaced with 100 *μ*L of complete culture media with 10% of MTT (5 mg/mL), and cells were incubated for 4–6 h. Finally, 100 *μ*L of DMSO was added in order to lyse the cells and release and solubilize the Formazan crystals. After 10 min of incubation at 37°C, absorbance was measured at 550 nm. Also the half maximal inhibitory concentration (IC_50_) was calculated by plotting viability versus log (concentration) and analyzed through the GraphPad Prism 6 software by nonlinear regression (Sigmoidal curve fit). In some experiments and after treatments, remaining viable cells were treated again with peptides. Cells were first treated with peptides for 24 h and washed carefully and then fresh culture medium was added and cells were further incubated for 72 h. Then peptides were added for the second time and incubated for 6 h and cell viability was quantified by the MTT assay and calculated as the percentage of average absorbance of each treatment relative to the average absorbance of the negative control.

### 2.4. Membrane Permeability Assay

The disruption of the cell membrane was assessed by propidium iodide (PI) uptake. CAL27 and SCC15 cells were cultured in 24-well plates and after adherence to the culture dish, the medium was replaced with FBS-free medium and the peptide was added. Cells were incubated for 1 h and then detached by enzymatic treatment with trypsin and resuspended in PBS with PI (1 *μ*g/mL) for 10 min (incubation in the dark). The fluorescent cells were analyzed by flow cytometry. STA (1 *μ*g/mL) and Triton X-100 (T-X100, 1% v/v) were used as controls.

### 2.5. Apoptotic/Necrotic Assay

1 × 10^5^ CAL27 and Het-1A cells were treated with peptides for 1 h at 37°C and then labeled with Annexin V-FITC/PI, using the Dead Cell Apoptosis Kit for flow cytometry (Thermo Fisher Scientific). As a control for necrosis, the cells were treated for 5 min with 0.2% T-X100. As a control for apoptosis, the cells were treated with 10 *μ*M of STA for 3 h; also the caspase activity was inhibited by 20 *μ*M Z-VAD-FMK for 2 h.

### 2.6. Hemolytic Activity Assay

5 mL of heparinized peripheral blood was centrifuged at 1000 g for 7 min. The erythrocyte-rich fraction was resuspended in 10 mL of PBS and washed twice by centrifugation at 1000 g for 7 min. The erythrocytes (2% hematocrit in PBS) were incubated with the LfB protein, LfcinB25, or the LfcinB25-derived peptides (125 *μ*g/mL in all cases), for 2 h at 37°C. PBS was used as negative control, while Tween-20 (20% v/v) in PBS was used as positive controls. The 96-well plates were centrifuged, the supernatants were collected, and the absorbance was determined at 540 nm.

### 2.7. qRT-PCR Analysis

Total mRNA was isolated by the trizol/chloroform method, and 1 *μ*g was treated with DNAse I (Invitrogen) and used for the reverse transcription reaction using the High Capacity cDNA Reverse Transcription kit (Applied Biosystems). The resulting cDNA was diluted 1 : 4 and assessed by PCR using Power Syber Green Master Mix (Applied Biosystems). Measurements were done in a 7500 Real Time PCR system. For each sample, PCR reactions were done in triplicate. The fold change in gene expression of cytokeratins 18 and 19, E-cadherin, vimentin, and ZEB-1 and ZEB-2 was calculated as the relative expression of the gene of interest to the expression of GADPH using the 2^−ΔCT^ method. Primers sequences used are shown in Supplementary Table  1 in Supplementary Material available online at http://dx.doi.org/10.1155/2015/630179.

### 2.8. Statistical Analysis

The results were expressed as the arithmetic mean values ± s.e.m. Comparisons between groups were performed using analysis of variance (ANOVA) followed by Dunnett's or Tukey's test, after verification of the assumptions of the statistical model comparisons. Comparison of means was performed using Student's *t*-test. For all statistical analyses, *p* values <0.05 were considered to be significant (*n* = 3).

## 3. Results

### 3.1. Cytotoxic Activity

**Figure 1:**
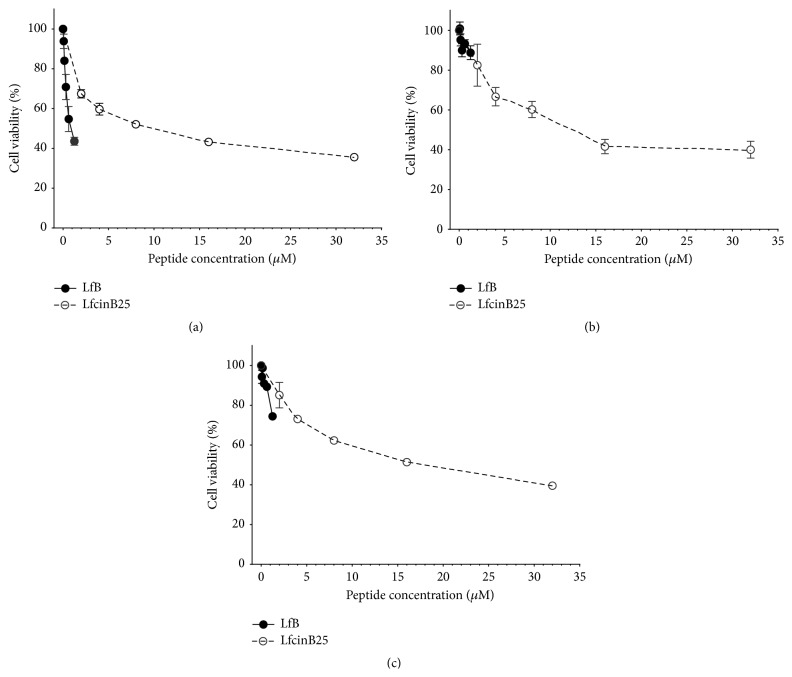
Cytotoxic effect of LfB and LfcinB25 in the OSCC tumorigenic cell lines CAL27 (a) and SCC15 (b) and the immortalized nontumorigenic keratinocytes cell line Het-1A (c). The cells were incubated for 24 h with the LfB protein and the LfcinB25 peptide. After treatment, cell viability was determined by MTT assay and calculated as the percentage of average absorbance of each treatment relative to the average absorbance of the negative control. The maximum concentration of the LfB protein was 1.25 *μ*M (100 *μ*g/mL) and of LfcinB25 32 *μ*M (100 *μ*g/mL). Each treatment was done in triplicate.

MTT cell viability assays showed that LfB was cytotoxic to CAL27 cells in a dose-dependent manner, showing 56.6% cytotoxicity at the highest concentration tested (1.25 *μ*M equivalent to 100 *μ*g/mL) (Figure 1(a), solid line). Only a 11.2% cytotoxicity was detected at the same concentration in the SCC15 cell line (Figure 1(b), solid line). In the nontumorigenic cell line Het-1A, the LfB cytotoxicity was 25.5% at the maximum concentration tested (Figure 1(c), solid line). Since it has been suggested that the LfB cytotoxic effect relies mainly on the N-terminal region of the protein, the LfcinB25 peptide was synthesized and its cytotoxic activity determined by the MTT assay. This N-terminal linear peptide exhibited a similar and dose-dependent cytotoxic effect both in OSCC cell lines (64.4% and 60% for CAL27 and SCC15, resp.) and in the nontumorigenic cell line Het-1A (60.5%) at the highest concentration tested (32 *μ*M, equivalent to 100 *μ*g/mL) (Figure 1, dotted line).

**Figure 2:**
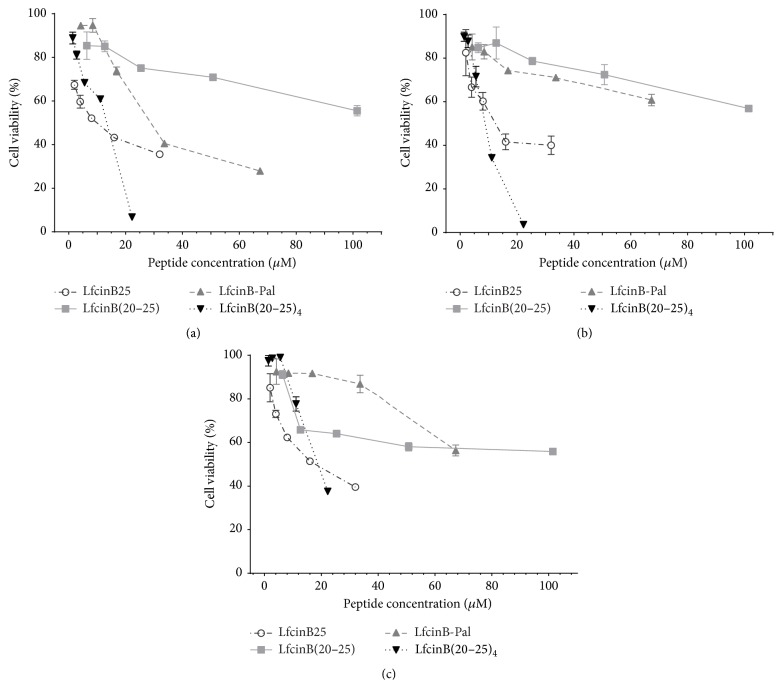
Cytotoxic effect of LfcinB-derived peptides in the OSCC tumor cell lines CAL27 (a) and SCC15 (b) and the immortalized nontumorigenic keratinocytes cell line Het-1A (c). The cells were incubated for 24 h with the peptides and cell viability was determined by the MTT assay and calculated as the percentage of average absorbance of each treatment relative to the average absorbance of the negative control. The maximum concentration of the peptides used was LfcinB25, 32 *μ*M; LfcinB(20–25), 101.5 *μ*M; LfcinB-Pal, 67.3 *μ*M; LfcinB(20–25)_4_, 22.25 *μ*M (all equivalent to 100 *μ*g/mL). The data are expressed as the mean ± s.e.m. (*n* = 3). LfcinB(20–25)_4_ *cf* LfcinB(20–25) had statistical significant differences at high concentration (100 *μ*g/mL) (ANOVA, posttest Tukey, *p* < 0.05).

New LfcinB25-derived peptides containing the RRWQWR motif were therefore designed and synthesized (peptides LfcinB-1 to LfcinB-4, Table 1). Some of them exhibited cytotoxic activity in the OSCC cell lines but they did not improve the results obtained with LfcinB25 (data not shown). Additional peptides based on this sequence were synthesized and tested for their cytotoxic activity (LfcinB(20–25), LfcinB-Pal, and LfcinB(20–25)_4_, Table 1). All of them exhibited cytotoxic activity in a dose-dependent manner (Figure 2). The monomer LfcinB(20–25) exhibited cytotoxicity in all cell lines but did not exceeded LfcinB25's effect despite the higher molarity used, nor was it selective against the tumorigenic cell lines. LfcinB-Pal, synthesized to increase the partial cationic charge and hydrophobicity, clearly improved the cytotoxic effect in CAL27 cells but only slightly in SSC15 cells. It is of note that the effect in the nontumorigenic cell line Het-1A was low even at 40 *μ*M. The strongest cytotoxic effect was obtained with the tetrameric peptide LfcinB(20–25)_4_. Some selectivity towards the tumor cell lines CAL27 and SCC15 was also observed in this case, with a cytotoxic effect reaching 93% and 96%, respectively, while in the immortalized nontumorigenic Het-1A cell line it was 62%. Additionally, it was found that a higher peptide concentration of LfcinB(20–25)_4_ is necessary to reach the IC_50_ in the cell line Het-1A compared with the OSCC cells lines (Table 2).

**Figure 3:**
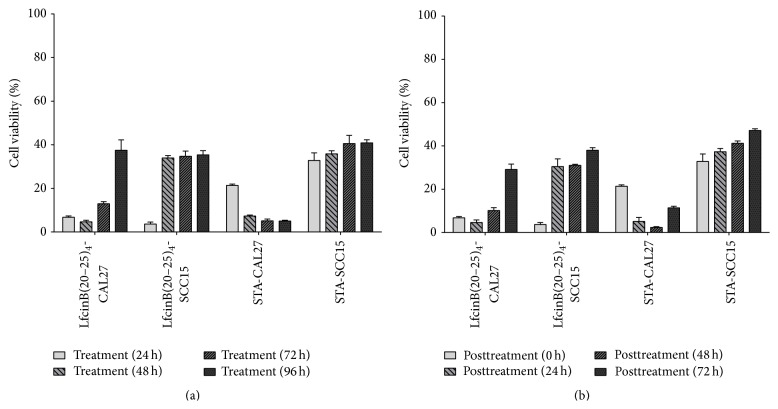
Cytotoxic activity of LfcinB(20–25)_4_ in long-term treatments. (a) SCC15 and CAL27 cells were incubated with the peptide for 24, 48, 72, and 96 h. (b) SCC15 and CAL27 cells were treated for 24 h with the peptide and washed, and cells were incubated for 0, 24, 48, and 72 h in fresh culture medium. After the treatments, cell viability was determined by the MTT assay and calculated as the percentage of average absorbance of each treatment relative to the average absorbance of the negative control. Cell viability was evaluated 24, 48, and 72 h after treatments and was calculated as the percentage of average absorbance of treatments in relation to average absorbance of negative control. The concentrations of LfcinB(20–25)_4_ and STA used were 22.25 *μ*M (100 *μ*g/mL) and 0.86 *μ*M (0.4 *μ*g/mL), respectively. Treatments were done in triplicate.

**Figure 4:**
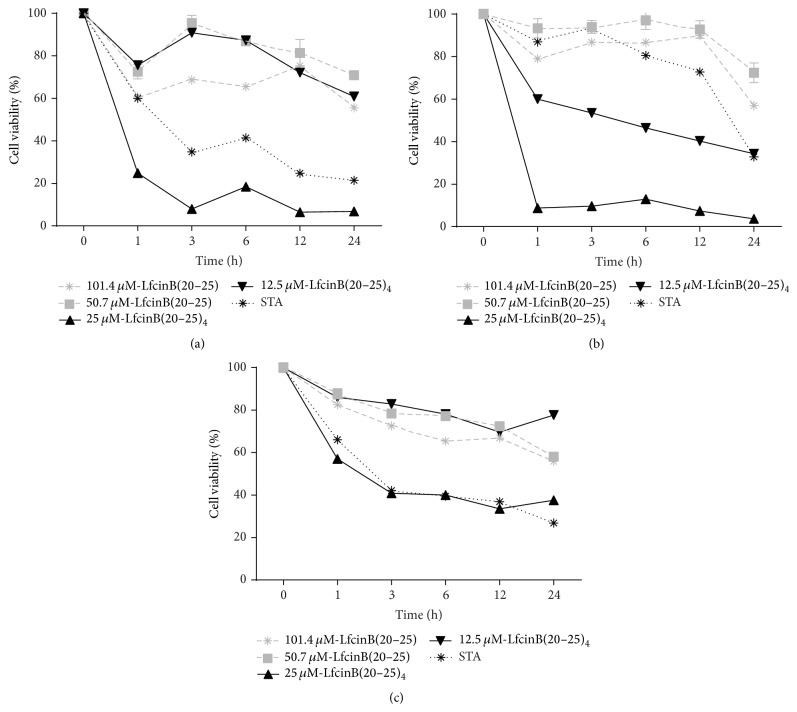
Cytotoxicity evaluation at different time points of treatment with LfcinB-derived peptides in the OSCC tumor cell lines CAL27 (a), SCC15 (b), and the immortalized nontumorigenic keratinocytes cell line Het-1A (c). The cells were incubated with LfcinB(20–25) or LfcinB(20–25)_4_ at the indicated times. After treatment, cell viability was determined by the MTT assay and calculated as the percentage of average absorbance of each treatment relative to the average absorbance of the negative control. The concentration of the LfcinB(20–25)_4_ used was 22.25 *μ*M (100 *μ*g/mL) and of LfcinB(20–25) was 101.5 *μ*M (100 *μ*g/mL). The STA concentration used was 0.86 *μ*M (0.4 *μ*g/mL) for CAL27 and 1.29 *μ*M (0.6 *μ*g/mL) for SCC15 and Het-1A. Each treatment was done in triplicate.

As has been previously observed with other therapeutic compounds, not all cells died after the various peptide treatments. The reasons for this are unknown and certainly varied. In order to explore this issue, we determined the proliferation capacity of the remaining cells after longer treatment periods with LfcinB(20–25)_4_ (Figure 3). The cytotoxic effect of LfcinB(20–25)_4_ in CAL27 was 98% after 24 h of incubation and progressively declined to 85% (after 72 h) and 62% (after 96 h) (Figure 3(a)). This progressive reduction was not observed in SCC15 cells, showing a rapid (from 24 h to 48 h period) recovery of viability (near 64%) which is maintained hereinafter; the reasons for this difference are unknown, but since SCC15 cells have a slower proliferation rate (42 h for SCC15 compared to 28 h for CAL27), this could explain in part the lack of progressive recovery; also the SSC15 cell line seems to have a higher resistance to STA treatment (Figure 3(a)). These results suggest that LfcinB(20–25)_4_ could exert its maximum effect before 24 h of treatment. In order to further investigate this, OSCC cells were treated for 24 h with the peptide as described previously, washed, and then cultured with fresh medium without peptides (Figure 3(b)). These results were very similar to those found in long-term treatments, confirming that its effect is produced before 24 h of treatment. In fact, LfcinB(20–25)_4_ exhibited a significant cytotoxic effect from the first hour of treatment in CAL27 (80%) and SCC15 (95%) (Figures 4(a) and 4(b)). A lower effect (42%) was found in the Het-1A cell line (Figure 4(c)) after 1 h of treatment with LfcinB(20–25)_4_ and this could not be differentiated from the STA treatment (Figure 4(c)); this suggests some specificity of the tetrameric peptide towards the tumorigenic cell lines.

### 3.2. Disruption of Cell Membrane

**Figure 5:**
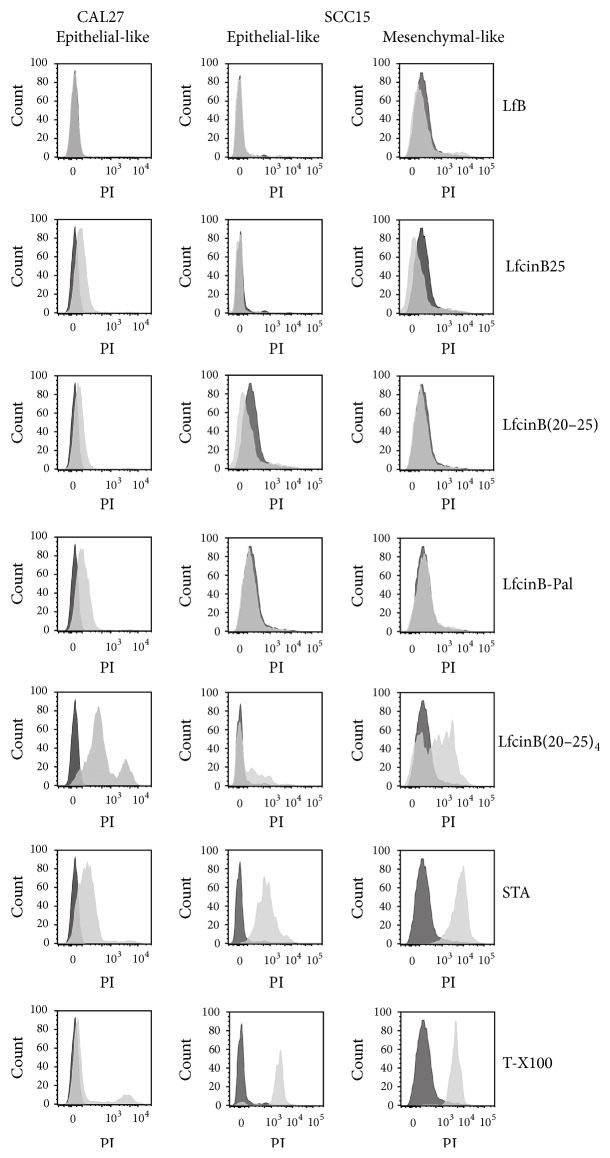
PI permeability in CAL27 and SCC15 cell lines evaluated by FACS. Cells were incubated with the different peptides for 1 h, after which they were harvested and incubated with PI in the dark for 10 min. Black plot: controls without treatment; gray plot: 1 hour of treatment with peptides, or 2.14 *μ*M (1 *μ*g/mL) STA control or 0.2% T-X100 control. The maximum concentration of the peptides used was 100 *μ*g/mL equivalent to LfcinB25, 32 *μ*M; LfcinB(20–25), 101.5 *μ*M; LfcinB-Pal, 67.3 *μ*M; and LfcinB(20–25)_4_, 22.25 *μ*M.

**Figure 6:**
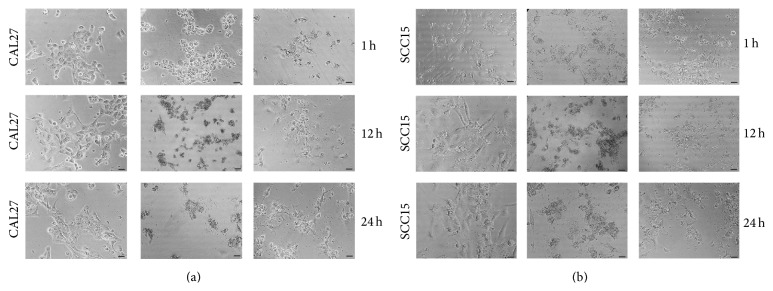
Cell morphology of CAL27 (a) and SCC15 (b) after treatment with LfcinB(20–25)_4 _for 1, 12, or 24 h. The STA concentration used was 0.86 *μ*M (0.4 *μ*g/mL) for CAL27 and 1.29 *μ*M (0.6 *μ*g/mL) for SCC15. Photomicrographs were taken with a phase-contrast microscope.

Given this rapid effect, the PI permeability in peptide-treated OSCC cells was evaluated (Figure 5). In the case of the SCC15 cell line, this evaluation was done for the two distinctive morphological cell populations observed in continuous culture independent of cell confluence (Supplementary Figure 1). These mesenchymal- and epithelial-like phenotypes were confirmed by the expression of phenotype-specific markers (Supplementary Figure 2). LfB, LfcinB25, and LfcinB(20–25) peptide had minimum effect, if any, on cell permeability in both cell lines. LfcinB-Pal has only a minimum effect in the CAL27 cell line, while it did not show any effect in the SCC15. On the contrary, LfcinB(20–25)_4_ exhibited an important lytic effect on both cell lines. On comparing the SCC15 cell phenotypes, the mesenchymal-like phenotype was found to be highly sensitive to the tetramer treatment (Figure 5). Interestingly, this effect was observed after only 1 h of treatment, which agrees with the previous cell viability assays (Figure 4). Morphological changes associated with LfcinB(20–25)_4_ treatment were seen almost immediately in terms of cell shrinkage in CAL27 and SCC15 from the first hour of treatment, while very few cells retained their morphology (Figure 6).

### 3.3. Mechanism Involved in the Cytotoxic Effect of LfcinB(20–25)_4_

**Figure 7:**
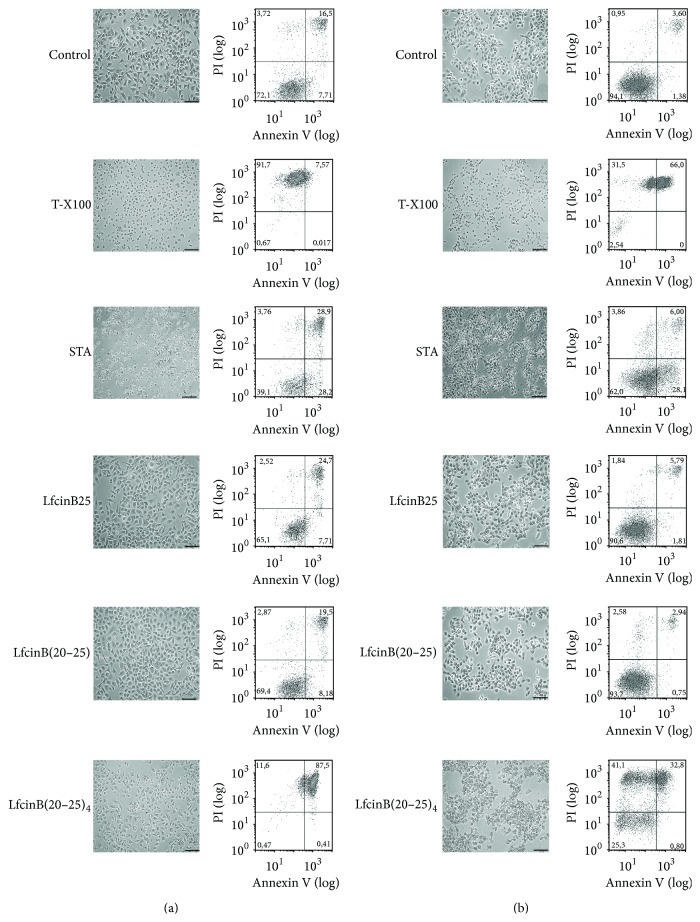
Assessment of necrosis/apoptosis in nontumorigenic cell line Het-1A (a) and the tumorigenic cell line CAL27 (b). Cells were detached and incubated with different peptides indicated for 1 h, after which they were labeled with both Annexin V-FITC and PI, and analyzed by flow cytometry. 10 *μ*M (4.66 *μ*g/mL) STA and 0.2% T-X100 were used as controls. The maximum concentration of the peptides used was 100 *μ*g/mL equivalent to LfcinB25, 32 *μ*M; LfcinB(20–25), 101.5 *μ*M; LfcinB-Pal, 67.3 *μ*M; and LfcinB(20–25)_4_, 22.25 *μ*M. Photomicrographs were taken with a phase-contrast microscope. Barr = 100 *μ*m.

In order to determine whether the rapid disruptive effect of the LfcinB(20–25)_4_ peptide is triggered by an apoptotic or necrotic process, the CAL27 tumorigenic and the Het-1A nontumorigenic cells were treated with the tetrameric peptide for 1 h, labeled with both Annexin V-FITC and PI, and analyzed by flow cytometry. The LfcinB(20–25)_4_-treated cells were significantly permeabilized (cell^PI+^/cell^PI+/Annexin+^) and an apoptotic cell population was not detected (cell^PI−/Annexin+^). A similar effect was found when the cells were treated with T-X100 (Figures 7(a) and  7(b)), indicating that the mechanism associated with the cytotoxic effect of the LfcinB(20–25)_4_ peptide is due to a necrotic event. Additionally, cells were treated with the caspase inhibitor Z-VAD-FMK and no differences were found (Supplementary Figure 3). These results showed that both LfcinB25 and LfcinB(20–25)_4_ have a cytotoxic effect possible due to a necrotic event (Figure 7). The necrotic damage was equivalent to the cytotoxicity found in the viability cell assays (Figure 2).

### 3.4. Hemolysis Test

With the aim of evaluating whether the peptides could cause a hemolytic effect, normal human erythrocytes were treated with LfcinB25 or the LfcinB25-derived peptides. The results showed that, at the maximum molar concentrations used in the cytotoxic assays (equivalent to 100 *μ*g/mL), the peptides did not exert any hemolytic effect (Supplementary Figure 4). The tetramer LfcinB(20–25)_4_ showed lysis of erythrocytes only at a concentration of 500 *μ*g/mL (data not shown).

### 3.5. Stability of the Peptides

Since the LfcinB(20–25)_4_ peptide has a significant cytotoxic effect in OSCC cells after 1 h of treatment and considering that no differences in longer treatment periods were found (Figure 3), we tested whether a second addition of the peptide could be cytotoxic. CAL27 cells were treated with LfcinB(20–25)_4_ peptide for 24 h, and then the medium was removed and fresh medium was added. Cells were incubated for additional 72 h at 37°C, and then a second dose of the same peptide was added for additional 6 h. The viability was quantified relative to cells treated with vehicle alone (Supplementary Figure 5). Interestingly, cytotoxicity was reduced to the same extent as the one obtained after the first treatment, suggesting that after 1 h of incubation the peptide availability is reduced, probably by rapid consumption, degradation, aggregation, or other undetermined effect. However this issue requires further investigation.

## 4. Discussion

LfcinB25 and its derived peptides may offer a therapeutic alternative for the treatment of OSCC, with higher selectivity towards cancer cells [41, 46, 48], an important advantage compared to the standard treatments [49, 50]. In the present study, we tested the cytotoxic activity of LfB, LfcinB25, and LfcinB25-derived peptides in the OSCC cell lines CAL27 and SCC15, using the Het-1A cell line (a nontumorigenic cell line) as control. Our results showed that the LfcinB25 peptide has similar effect to the LfB protein in OSCC cells. Therefore, short LfcinB25-derived peptides were synthesized based on the fact that they should have amphipathic characteristics similar to LfcinB25, properties associated with the presence of the cationic arginine and the hydrophobic tryptophan residues, a hallmark of LfcinB antimicrobial activity [34, 48, 51]. These peptides showed some cytotoxic activity in the OSCC cell lines but they did not improve the results obtained with LfcinB25 (data not shown). Peptides having cytotoxic activity in the same range as LfcinB25 included the RRWQWR motif (amino acids 20 to 25 of LfcinB25), a sequence that has shown reduced cytotoxicity in hematological malignancies [41, 52]. However, intracellular delivery of LfcinB(20–25) via fusogenic liposomes results in a potent cytotoxic activity that involves the action of caspases and cathepsin B without requiring ROS generation [52]. Also, a chimerical peptide containing 7 Arg residues linked by a Gly residue to LfcinB(20–25) exhibits a membranolytic effect after 30 min of treatment in the T-cell leukemia and B-cell lymphoma cell lines, but not in normal activated T-cells [42]. It has been suggested that the positive charges achieved in this way could favor the interaction with the negatively charged surface of cancer cells, whereas the hydrophobic amino acids of LfcinB(20–25) could subsequently permeabilize the cell membrane [44].

In agreement with these reports and our own results, we designed a peptide by increasing the number of hydrophobic amino acids while maintaining the net positive charge (+3) and keeping the RWRQWR sequence, that is, LfcinB-Pal (RWQWRWQWR). The LfcinB-Pal peptide showed an improved cytotoxicity in OSCC cell lines compared to LfcinB(20–25), especially in the CAL27 cells.

Also, it has also been shown that dendrimeric peptides could be used as a novel drug delivery system, maximizing efficacy [53]. Therefore, a tetrameric peptide, LfcinB(20–25)_4_, was designed in order to increase the hydrophobicity and the positive charges (net positive charge of +16), keeping the sequence RRWQWR. Our results showed that the cytotoxic effect of this peptide was dramatically improved in both CAL27 and SCC15 cells after 24 h of treatment, with 93% and 96% inhibition, respectively, significantly improving the cytotoxic effect of LfcinB25 at lower molar concentrations. For Het-1A cells, the cytotoxicity of LfcinB(20–25)_4_ was 63%, which is significantly lower than the effect in tumorigenic cells, but significantly higher in comparison with the negative control (cells without treatment). The fact that premalignant cells are less affected by the tetrameric LfcinB(20–25)_4_ peptide than the tumorigenic ones is suggestive of a possible reduced effect in normal cells, but this deserves further experimentation. Since immortalization is an important step in carcinogenesis [54], a cytotoxic effect on the Het-1A cell line could be anticipated, as was the case here. However it is interesting to note that no lytic effect of LfcinB(20–25)_4_ was observed in normal red blood cells.

Few studies deal with the fact that there are always a few cells refractory to the drug being tested. Here, we have shown that these few cells were able to proliferate and that they may represent a cell subpopulation resistant to treatment. This has been previously reported, and it has been suggested that these cells having a more mesenchymal phenotype are more invasive and responsible for the metastatic process [55]. Cell viability recovery after peptide treatment was very fast but limited in SCC15 cells while it was progressive in CAL27, a result consistent with the latter being more epithelial. Additionally, SCC15 cells were more resistant to treatment with peptide or STA, but in this case the mesenchymal phenotype was not responsible for this effect, since we demonstrated that membrane permeability was severely affected in this subpopulation compared with the more epithelial one.

The cytotoxic mechanism associated with LfcinB25 and LfcinB25-derived peptides is still unclear and could be dependent on the type of cancer cell evaluated [40, 42, 45, 52, 56]. It has been shown that LfcinB25 may induce different types of cell death: in some types of leukemia and gastric cancer cell lines, cytotoxicity is determined by caspase-dependent apoptosis or autophagy [40, 41, 56], while in fibrosarcoma, neuroblastoma, and other blood malignancies, a cytolytic mechanism has been proposed [36, 42, 43]. Consistent with this, it has been proposed that LfcinB25-derived peptides could interact with the cell membrane and cause its subsequent disruption via a mechanism similar to the one described for pathogenic microorganisms [43, 44, 57, 58]. We determined that the cytotoxic effect of LfcinB(20–25)_4_ in OSCC cells was fast, causing significant damage to the cell membrane after 1 h of treatment, triggering cell necrosis. This rapid effect is relevant since we have also shown that LfcinB(20–25)_4_ peptide availability could be an important issue. Our results indicate that severe damage to cell membrane permeability is caused by the LfcinB(20–25)_4_ peptide and that this tetrameric peptide exhibits partial selective cytotoxicity towards tumorigenic cells lines. Our results suggest that the tetramer here evaluated could be considered as a novel therapeutic agent useful in the treatment of OSCC.

## Supplementary Material

Supplementary Material shows photomicrographs of the OSCC cell lines at different culture confluence; expression of epithelial and mesenchymal markers in both SCC15 cell subpopulations; inhibition of apoptosis by the caspases inhibitor Z-VAD-FMK; evaluation of the hemolytic effect of LfcinB25 derived peptides, and the effect of a second addition of the tetrameric peptide on OSCC cell viability.

## Figures and Tables

**Table 1:** LfcinB-derived peptides used in this study.

Peptide	Amino acid sequence^1^	Charge
LfcinB-1	F**K**A$\underline{RRWQWR}$M	+4
LfcinB-2	$\underline{RRWQWR}$M**KK**LG	+5
LfcinB-3	$\underline{RRWQWR}$M**RR**LG	+5
LfcinB-4	F**K**C$\underline{RRWQWR}$M**KK**LGA	+6
LfcinB(20–25)	**RR**WQW**R**	+3
LfcinB-Pal	**R**WQW**R**WQW**R**	+3
LfcinB(20–25)_4_	(**RR**WQW**R**)_4_-**K**_2_-(Ahx)_2_-C_2_	+12
LfcinB25	F**K**C**RR**WQW**R**M**KK**LGAPSITCV**RR**AF	+8

^1^Positively charged amino acids are shown in bold.

**Table 2:** IC_50_ of LfcinB-derived peptides.

Cell line	Peptide	IC_50_ (*µ*M)		
		1 hour	3 hours	24 hours
CAL27	LfcinB25	5.248 ± 1.54	9.694 ± 1.38	8.67 ± 1.44
	LfcinB(20–25)	>101.4	>101.4	>101.4
	LfcinB-Pal	22.77 ± 1.12	22.6 ± 1.02	21.54 ± 1.05
	LfcinB(20–25)_4_	16.01 ± 1.36	17.44 ± 1.77	9.016 ± 1.38

SCC15	LfcinB25	>32	>32	4.04 ± 2.87
	LfcinB(20–25)	>101.4	>101.4	>101.4
	LfcinB-Pal	>67.26	>67.26	>67.26
	LfcinB(20–25)_4_	13.58 ± 1.06	12.58 ± 1.06	9.048 ± 1.07

Het-1A	LfcinB25	>32	4.127 ± 1.20	8.12 ± 1.20
	LfcinB(20–25)	>101.4	>101.4	>101.4
	LfcinB-Pal	>67.26	62.19 ± 1.56	>67.26
	LfcinB(20–25)_4_	>24.96	18.79 ± 1.11	17.37 ± 1.11
