# Supplementary material for: A Tetrameric Peptide Derived from Bovine Lactoferricin Exhibits Specific Cytotoxic Effects against Oral Squamous-Cell Carcinoma Cell Lines
Source: Biomed Res Int. 2015 Nov 2;2015:630179. doi: 10.1155/2015/630179 (PMC4644816; doi:10.1155/2015/630179)
Supplement: Supplementary file 1 — Supplementary Material shows photomicrographs of the OSCC cell lines at different culture confluence; expression of epithelial and mesenchymal markers in both SCC15 cell subpopulations; inhibition of apoptosis by the caspases inhibitor Z-VAD-FMK; evaluation of the hemolytic effect of LfcinB25 derived peptides, and the effect of a second addition of the tetrameric peptide on OSCC cell viability. [file 630179.f1.pdf]

SUPPLEMENTARY FIGURES AND TABLE

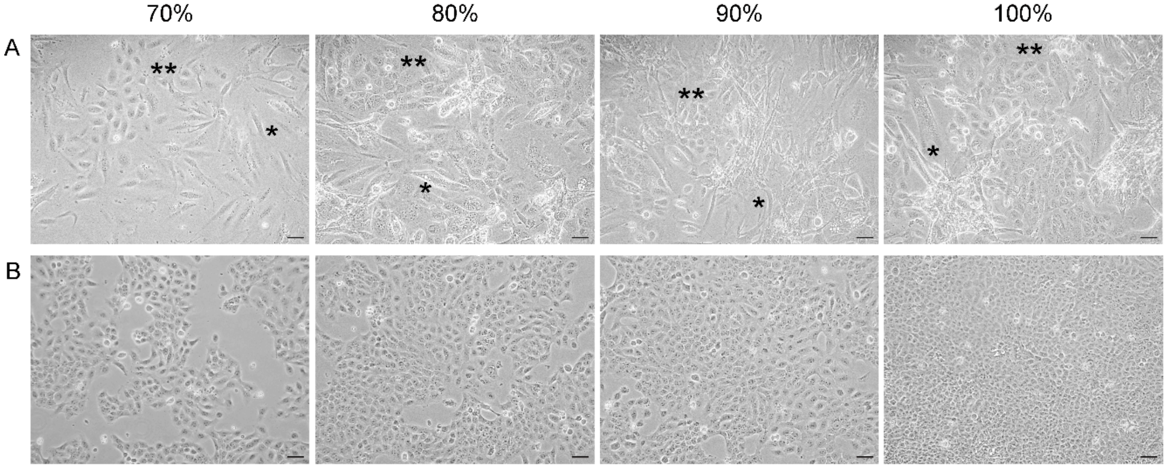

SUPPLEMENTARY FIGURE 1

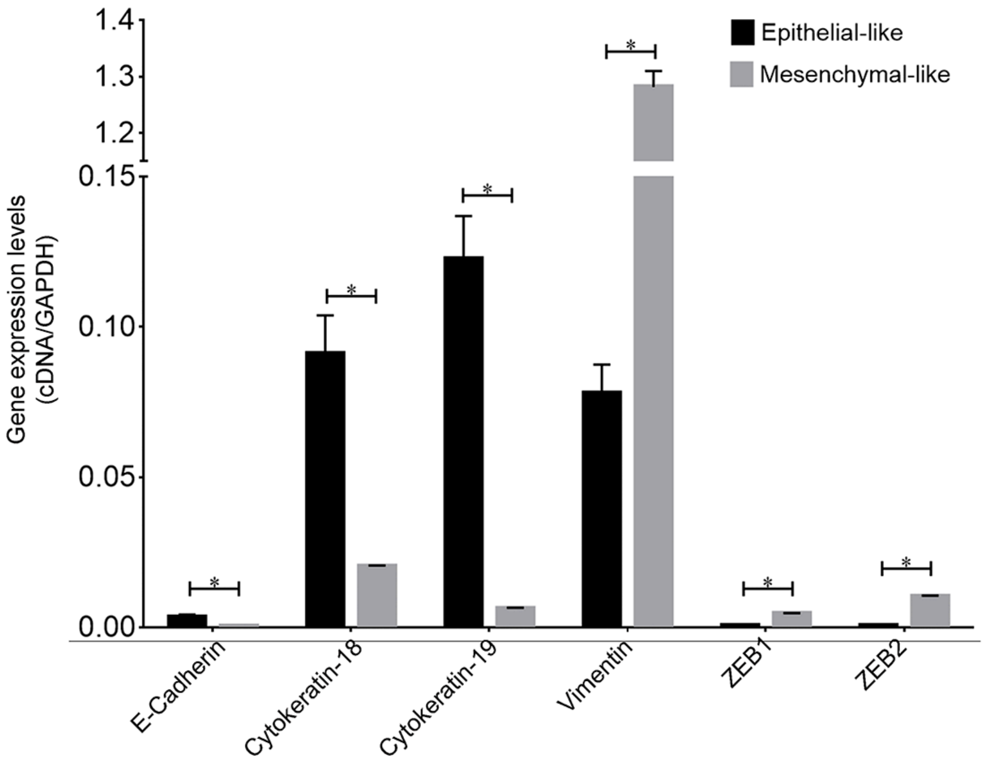

SUPPLEMENTARY FIGURE 2

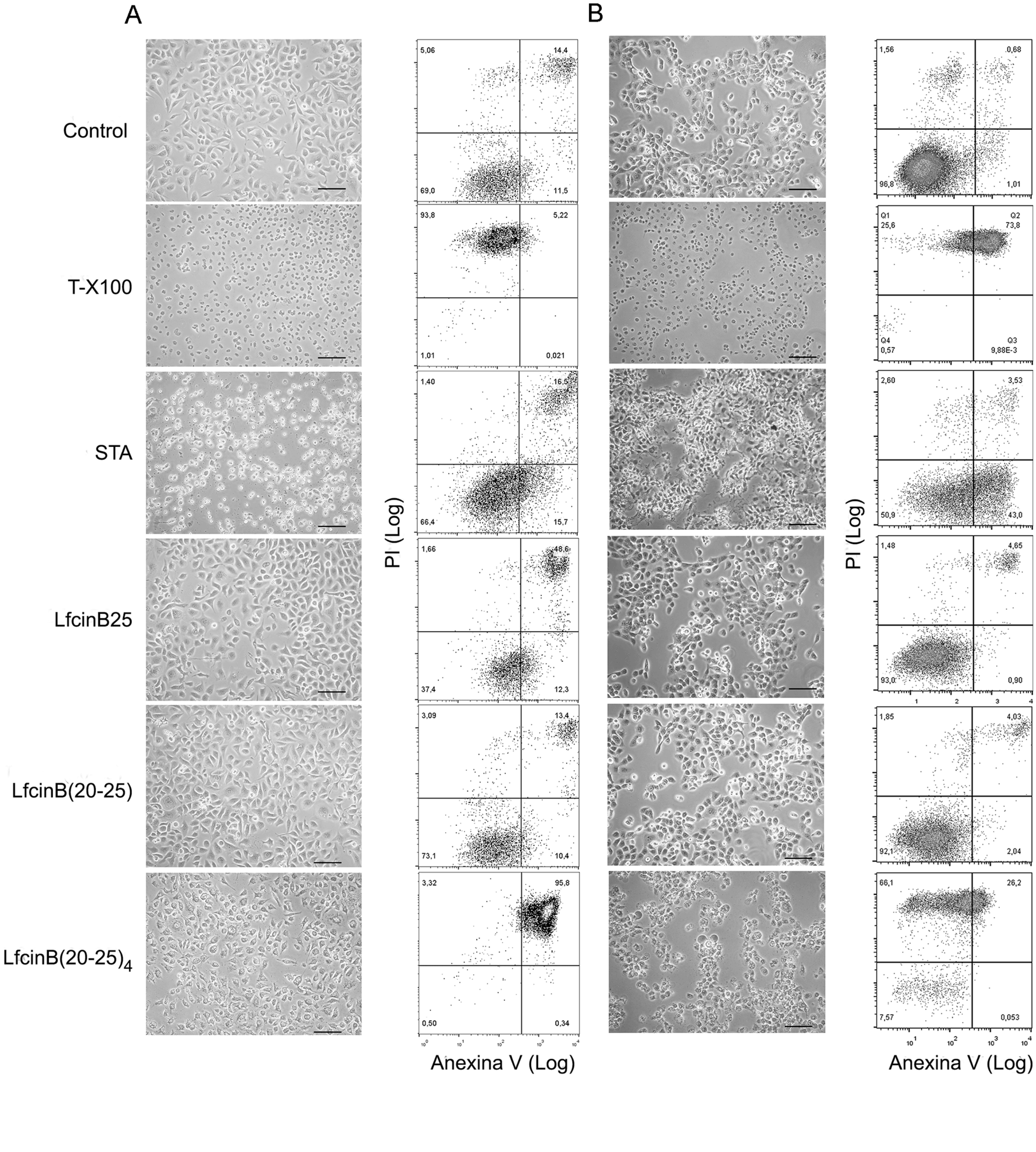

SUPPLEMENTARY FIGURE 3

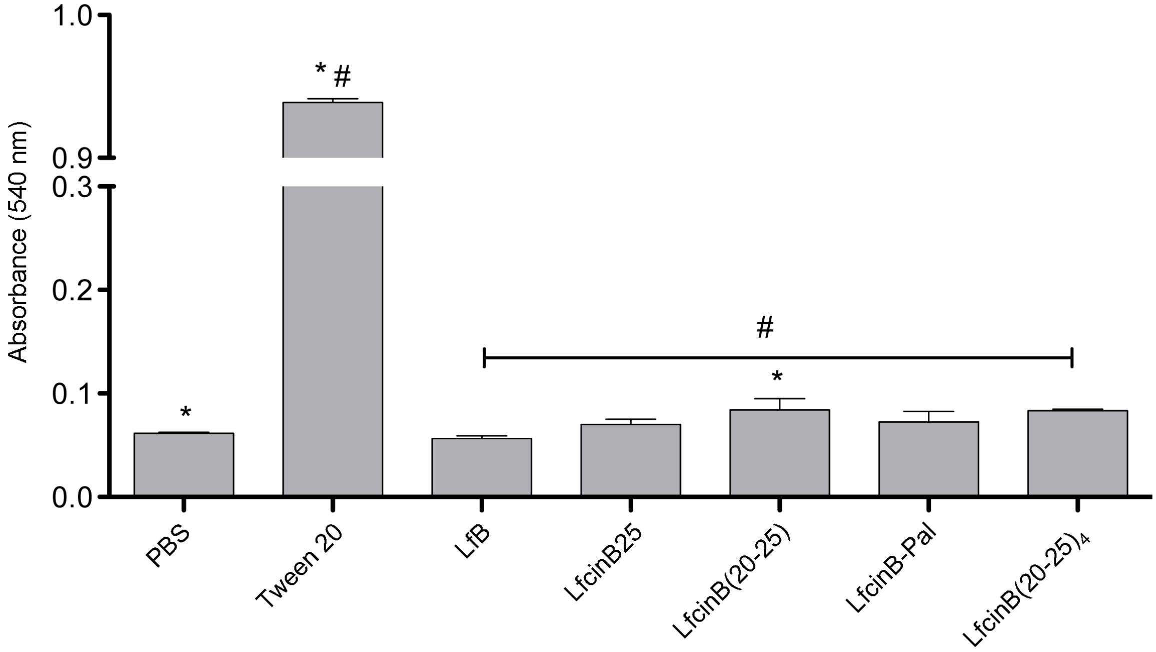

SUPPLEMENTARY FIGURE 4

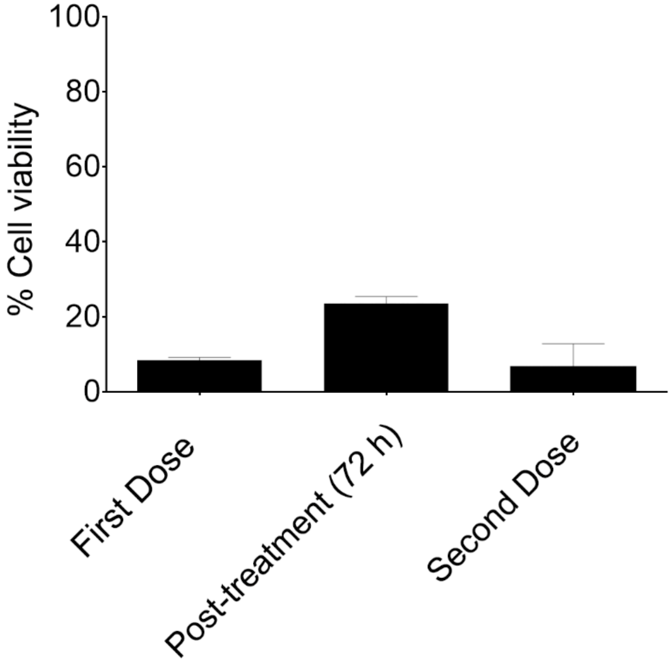

SUPPLEMENTARY FIGURE 5

Supplementary Table 1. Primers used in this study

Gen	Forward primer	Reverse primer
CYTOKERATIN 18	AGCTCAACGGGATCCTGCTGCACCTTG	CACTATCCGGCGGGTGGTGGTCTTTTG
E-CADHERINA	TGGACAGGGAGGATTTTGAG	ACCCACCTCTAAGGCCATCT
CYTOKERATIN 19	GAGCATGAAAGCTGCCTTGG	GGGCTTCAATACCGCTGATC
VIMENTIN	CGAGGACGAGGAGAGCAGGATTTCTC	GGTATCAACCAGAGGGAGTGA
ZEB1	AAGAATTCACAGTGGAGAGAAGCCA	GGTTTCTTGCAGTTTGGGCATT
ZEB2	TGTAGATGGTCCAGAAGAAATG	CCATTGTTAATTGCGGTCT

Legends to supplementary figures

Supplementary Figure 1. Photomicrographs of the cell lines CAL27 and SCC15 at different culture confluence. A) SCC15 cell line: the * show the cells with epithelial-like morphology while the ** point to cells with mesenchymal-like morphology; B) CAL27 cell line. The percentage of cell confluence is indicated. Photomicrographs were taken with a phase contrast microscope. Barr = 100 µm.

Supplementary Figure 2. Expression of epithelial and mesenchymal markers in SCC15 cell subpopulations. The mesenchymal- and epithelial-like separately cultured cells were harvest and tested for E-cadherin, cytokeratins 18/19, vimentin, and ZEB-1-and -2 expression. The results were normalized to GADPH expression and specific gene expression was calculated using the $2^{\Delta ct}$ method. The results were expressed as the arithmetic mean values ± standard error of a test done in triplicate. Statistical differences are show with *, (Anova, post-test Tukey, p <0.05).

Supplementary figure 3. Inhibition of apoptosis. Het-1A (A) and CAL27 (B) cells were treated with Z-VAD-FMK 20 µM and incubed for 2 h; then the cells were detached and $1x10^5$ cells were incubated with the different peptides at 37°C for 1 h, after which they were labeled both with Anexin V-FITC and PI. 10 µM (4.66 µg/mL) STA or 0.2% T-X100 were used as controls. The maximum concentration of the peptides used was 100 µg/mL equivalent to: LfcinB25, 32 µM; LfcinB(20-25), 101,5 µM; LfcinB-Pal, 67.3 µM; and $LfcinB(20\text{-}25)_4$, 22.25 µM. Photomicrographs were taken with a phase-contrast microscope. Barr = 100µm.

Supplementary Figure 4. Evaluation of the hemolytic effect. A 2% erythrocyte cell suspension was prepared and incubated for 2 h with the indicated peptides or controls. The hemolysis was quantified by indirect measurement of hemoglobin released into the culture medium by absorbance at 540 nm. Statistical comparison was done using Dunnett's test (p<0.05). *: statistical difference compared to PBS; # statistical difference compared to Tween 20.

Supplementary Figure 5. Testing $LfcinB(20\text{-}25)_4$ stability. CAL27 cells were treated with $LfcinB(20\text{-}25)_4$ for 24 h (first dose), incubated for 72 h at 37°C with fresh medium (post-treatment (72 h)) and then a new peptide treatment was performed (second dose), the cells were incubated for additional 6 h and the viability of the cells was quantified by MTT assay. (Anova, Post-test Tukey, p < 0.05): * Post-treatment (72 h) *cf* first or second dose.
